# Rehabilitation of Soccer Players’ Knee Injuries: Cartilage Reconstruction, Anterior Cruciate Ligament Surgery, and Intensive Recovery—A Pilot Study

**DOI:** 10.3390/jcm12216893

**Published:** 2023-11-01

**Authors:** Bartłomiej Kacprzak, Karolina Rosińska

**Affiliations:** 1 Orto Med Sport Łódź, 28 Pułku Strzelców Kaniowskich 45, 90-640 Łódź, Poland; hipokrates@op.pl; 2 Wolf Project Studio Krzysztof Król, ul. Gdańska 79/D01, 90-613 Łódź, Poland

**Keywords:** anterior cruciate ligament, cartilage, microfracture, Hyalofast, soccer players, rehabilitation

## Abstract

Knee injuries, particularly anterior cruciate ligament (ACL) damage and cartilage defects, are highly prevalent among athletes and affect their sports performance and long-term joint function. The purpose of this research was to evaluate the effectiveness of a comprehensive combination therapy approach for individuals with ACL and cartilage injuries. Twelve professional soccer players aged 18 to 30 years underwent bone–tendon–bone ACL reconstruction, microfracture cartilage repair surgery, and hyaluronic acid scaffold treatment. Early postoperative rehabilitation included immediate supervised physiotherapy and complete weight bearing. Follow-up assessments involved clinical evaluations, functional joint assessments, and magnetic resonance imaging (MRI) scans to measure cartilage defect repair and symptom alleviation. The results showed that patients resumed pain-free activities within 3–4 weeks and returned to their pre-injury level within 4.5 months. MRI demonstrated the absence of inflammatory reactions, repair of marrow edema, and the emergence of new cartilage. Six months and one year after surgery, the Knee Injury and Osteoarthritis Outcome Score (KOOS) and the Short Form (36) Health Survey (SF-36) questionnaire results demonstrated considerable improvement in patients’ health condition and quality of life. Overall, the study suggests that the combination of Hyalofast membranes, microfracture surgery, tissue adhesive, and intensive postoperative physical therapy may be a potential alternative to commonly used treatments for patients with ACL rupture, allowing them to recover efficiently and return to sports activities.

## 1. Introduction

Knee injury, including ACL and cartilage defects, is a common problem among athletes and physically active people (the frequency of significant cartilage loss in acute ACL injuries ranges from 16% to 46%) [1,2,3,4,5,6]. Such injuries are often seen in soccer players and can cause long-term inability to play [7]. Injuries of ACL are the predominant form of knee ligament injuries and these injuries can have a devastating impact regardless of the level of sports activity or age group [8]. However, studies have documented that the highest prevalence of ACL injuries occurs among athletes aged between 15 and 40 years [9]. Therefore, ACL reconstruction is one of the most frequently performed orthopedic procedures globally [2,10,11].

Cartilage defects are common problems in both knees of athletes and are less physically active. Damage to the articular cartilage often forces patients to limit or change their sports activities [12,13]. In the case of surgical treatment in athletes, repaired and reconstructed cartilage must be able to withstand the high mechanical loads characteristic of sports types [14]. Techniques used to repair cartilage, such as microfractures, osteochondral transplants (OATs) or allografts, and autologous chondrocyte implants (ACIs) have demonstrated that ACL reconstruction procedures effectively reduce pain and enhance knee function among athletes [13,15]. Furthermore, inexpensive techniques, such as microfracture, remain the most commonly used procedures for cartilage damage and show potential for return to sport at short-term follow-up [14,16,17,18]. This is primarily due to the fact that it is a relatively inexpensive and one-step arthroscopic procedure [19]. It is affordable and technically simple [20].

When compared to grafts, the primary advantage of microfractures lies in the reduced risk of infection and health issues at the donor site—as well as a decreased likelihood of requiring additional surgeries. However, less permanent fibrocartilage development occurs following microfractures. This method does not repair the bone and may compromise future cartilage surgery outcomes [20]. Although most patients have favorable to exceptional results after microfracture, up to twenty-five percent or more fail at the ten-year mark [21]. Consequently, to enhance the regenerative potential, biological scaffolds composed of a matrix of porcine collagen, polyglycolic acid, or hyaluronic acid have also been introduced into the cartilage defect following microfracture. This incorporation establishes a mechanically stable structure that facilitates the absorption of subchondral blood. The inserted matrix functions as a biocompatible and temporary scaffold, concentrating mesenchymal stem cells and growth factors specifically at the site of the defect—rather than dispersing them throughout the joint. This targeted approach promotes localized cartilage repair and the regeneration process more effectively [22,23,24].

Repairing cartilage defects with Hyalofast^TM^ scaffold (fibrin membrane stimulating cartilage growth) has emerged as a compelling alternative for patients experiencing intense joint pain [25,26,27,28]. Hyalofast is composed only of semisynthetic hyaluronic acid derivatives, which are natural components of the extracellular matrix and fundamental constituents of human cartilage. This substrate enables the differentiation of mesenchymal stem cells into chondrocytes, which are capable of producing physiological cartilage. Hyalofast, upon application, creates a chondroprotective layer that extends the survival of mesenchymal stem cells at the site of the cartilage defect following mobilization through the microfracture technique [25,26,27,29]. However, despite significant advances in the treatment of knee injuries, recovery time can be prolonged, which significantly delays the return of athletes to active sports. Therefore, preparation of a proper rehabilitation program is important for optimizing surgical outcomes. Rehabilitation protocols can significantly increase athletes’ physical activity and reduce the risk of reinjury [15]. Returning to sports is also limited by the biological healing process [30]. Postsurgical rehabilitation must consider that healing of cartilage following microfracture occurs in three biological phases: formation of clots, repair of cartilage growth, and cartilage maturation [31].

The clinical significance of our research is found in the setting of cartilage and ACL injuries in highly skilled athletes, notably professional soccer players. The low effectiveness of conservative treatment for high-grade cartilage injuries and the desire to quickly return to the game prompted us to look for alternative treatment methods [28]. The main hypothesis of our study posits that an intensive rehabilitation program, initiated one day after ACL reconstruction surgery combined with cartilage repair using microfractures and a hyaluronan scaffold (Hyalofast), will yield superior outcomes for professional athletes, enabling them to achieve a rapid return to sports at the pre-injury level.

The objective of our research was to evaluate the efficacy of this comprehensive treatment approach specifically tailored for professional athletes, who require swift recovery and restoration of functional performance to resume their athletic pursuits at the highest level. What distinguishes our study from others that were published is the early implementation of a complete physiotherapy program, beginning on the first day following surgery. This method provides quicker recovery and early full-load weight-bearing of the operated limb. In the case of professional athletes, emphasis on a comprehensive rehabilitation strategy is crucial because it promotes their return to sports without compromising long-term outcomes. The presented series of cases in this pilot study suggests the potential efficacy of the applied therapeutic procedure in patients with defects of the ACL and knee joint cartilage.

## 2. Materials and Methods

### 2.1. Patient Selection

The study inclusion criteria were active soccer players with focal damage to grade IV according to The International Cartilage Repair Society (ICRS) [32] and ACL injuries who required surgery, while also having no history of cartilage surgery on the affected knee joint. Conversely, patients under 18 years old or over 40 years old and those with muscle disorders such as myasthenia gravis, progressive malnutrition, and periodic paralysis were excluded. Patients who were unwilling to co-operate with the treatment or refused supervised rehabilitation in the clinic were also excluded. Additionally, patients with abnormal bleeding or coagulation function, incomplete follow-up, imaging data, or a follow-up time of less than six months were not eligible for inclusion.

This study was conducted retrospectively. The patients were identified from the clinical database [32]. A cohort of 12 male individuals (18–30 years old; mean age—26 ± 4 years) who were active professional soccer players underwent one-stage knee cartilage surgery accompanied by primary reconstruction of the ACL. All patients had lesions in the medial femoral condyle (MFC) (100%) as a result of a traumatic injury sustained during a soccer match (100%). The average duration of follow-up in the study was 19.75 (± 6.79, range 14–24) months. Table 1 provides comprehensive characteristics of the patients included in this study.

The patients in this study underwent a one-stage surgical intervention to address both ACL reconstruction and cartilage regeneration using a combination of Hyalofast membranes (Anika Therapeutics Inc., Bedford, MA, USA), microfractures, and tissue glue stabilization. Before surgery, a comprehensive physical examination and MRI evaluation were performed. During the examination, the patients presented with severe effusion, tenderness, and restricted range of movement in the affected knee joint. The Lachmann and pivot shift tests were positive. After MRI (SIGNA 1.5T HDx; GE HealthCare Technologies Inc., Chicago, IL, USA) confirmation of an ACL tear, evaluation of the size and location of the lesion, evaluation of pain intensity, and verification of conservative treatment failure, the attending physician referred the patients for surgery. All the patients who participated in this study provided written informed consent.

### 2.2. Operation Procedure

**Figure 1:**
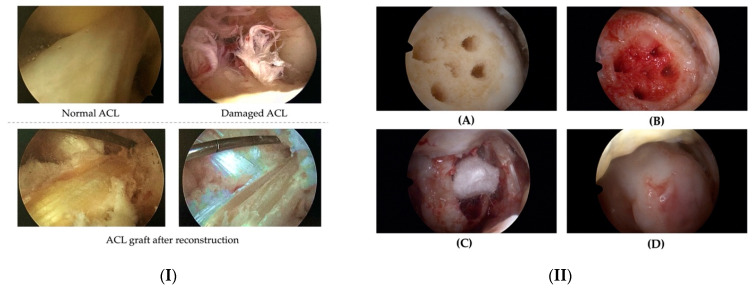
ACL arthroscopy images: normal, injured and rebuilt with a prepared ACL graft (**I**) and the cartilage defect site (**II**): Microfracture was performed at the bottom (**A**), the site was then filled with patient blood (**B**), a Hyalofast membrane was implanted (**C**) and stabilised with tissue adhesive (**D**).

All surgical procedures were performed by the same surgical team and surgeon under general anaesthesia. The first step was to implant anteromedial and anterolateral portals, followed by a complete examination of the joint structures, including ligaments, menisci, and the type and depth of cartilage injury. The bone–tendon–bone (BTB) technique was used to rebuild the ACL, with the patellar ligament serving as a graft (Figure 1(I)). The graft was taken from the patellar tendon with two bone blocks (one from the patella and one from the tibia) and prepared outside the patient. During graft preparation, the Arthrex RetroConstruction Tibial Guide (Arthrex, Naples, FL, USA) targeting device was used to build femoral and tibial channels for the future ligament. The prepared graft was inserted and fixed. Titanium screws were used in the femoral and tibial bone channels to increase the bond strength.

Following careful removal of the damaged tissue, the size of the cartilage defect was assessed, and microfracture was performed at the base of the defect using specialized instruments (Figure 1(IIA)). Prior to implantation of the Hyalofast membrane, the site was cleaned for maximum visibility. The cartilage defect was subsequently filled with the patient’s blood (Figure 1(IIB)) and a Hyalofast scaffold was inserted through a cannula and spread over the surface with a needle and probe (Figure 1(IIC)). After stabilising the Hyalofast, the defect was filled with the patient’s blood sample, and TISSEEL Lyo tissue glue (Baxter International Inc., Deerfield, IL, USA) was used to fix the Hyalofast implant (Figure 1(IID)).

After surgery, patients received anticoagulant medication (Neoparin 0.4/0.6; SciencePharma sp. z o.o. sp.k., Warsaw, Poland) once daily for 20 days. Additionally, antibiotic medication (Clindamycin 600, every 8 h; MIP Pharma Polska Sp. z o.o., Gdansk, Poland) was administered every 8 h for 2 weeks. It should be noted that no joint punctures or drains were used during the duration of therapy.

### 2.3. Follow-Up Evaluation

This study aimed to evaluate the treatment outcomes of athletes by comparing their preoperative condition with a functional evaluation performed during follow-up visits. All patients completed the SF-36 and KOOS questionnaires before surgery and at 2, 6, 12, 24 weeks, and 1 year after surgery. KOOS is a composite tool comprising five subscales that evaluate the severity of pain (PAIN), symptoms (SYMPTOMS), function in daily living (ADL), function in sports and recreational activities (SPORT/REC), and quality of life (QOL) [33]. The SF-36 is used to assess the general health and well-being of patients and comprises eight domains that evaluate physical and mental function [34]. Additionally, cartilage regeneration was assessed using 1.5 T MRI scans after an average of 6–8 weeks, six months, and one year. MRI provides multidimensional evaluation of the joint with high sensitivity to soft tissue, enabling an accurate assessment of the cartilage and its remodelling process during follow-up [27,35].

### 2.4. Rehabilitation Protocol

No orthoses or external supports were used during the postoperative rehabilitation period. An advanced cooling system and compression stockings (GAME READY knee wrap (CoolSystems, Inc., Alpharetta, GA, USA), as well as a regeneration and massage system (Normatec (Hyper Ice, Inc., Irvine, CA, USA)) were used to treat edema and pain. On the first day after surgery, rehabilitation began with full weight-bearing assisted by elbow crutches, which were then removed seven days after the procedure, and the patient’s range of motion was not impaired. Resistance training, eccentric-concentric exercises, and full body weight exercises were included in the rehabilitation program, with a focus on strengthening the erector and flexor muscles of the knee and increasing the range of motion. Proprioception activities were also included in unstable environments. A physiotherapist supervised the exercise regimen, including manual approaches. The athletes began preliminary training sessions three weeks after surgery—under the supervision of a physiotherapist and a physician. The rehabilitation protocol presented in Table 2 was the same for all 12 subjects. This consistency in the rehabilitation approach was maintained to ensure standardized treatment for all participants. The duration of each stage of the rehabilitation process varied depending on individual progress and abilities. The protocol allowed for flexibility in adjusting the timing of progression through the stages to accommodate the unique needs of each patient. This approach ensured that patients could advance through the stages at their own pace, optimizing their rehabilitation experience. The number of repetitions within each training session also depended on individual patient progress. Rest intervals between training sessions were tailored to the specific requirements of each patient. These rest intervals were determined based on individual progress and the ability to recover effectively between sessions.

### 2.5. Statistical Analysis

Results from the KOOS and SF-36 questionnaires are presented as the mean value ± standard deviation (SD). The obtained data were analysed using one-way analysis of variance (ANOVA) to determine statistical significance. The results were considered significant if the *p*-value (*p*) was less than 0.05. If statistical significance was present, it was indicated by an asterisk in the tables.

## 3. Results

### 3.1. KOOS and SF-36 Scale

The results of patient evaluations, as evaluated by two separate questionnaires, the KOOS and SF-36, are provided below. The general results for all patients are shown in Table 3.

The analysis of KOOS and SF-36 questionnaire findings indicated a notable and statistically significant improvement in patients’ health status and quality of life at six months and one year after surgery, respectively. Significant improvements were observed in pain, symptoms, everyday activities, and sports-related indicators. In addition, a general increase in the quality of life (KOOS scale) was obtained. There was a substantial difference in the KOOS scores obtained before surgery and at 6 and 12 months postoperatively, demonstrating that the surgical technique was beneficial in improving the health status of patients. Similarly, a significant difference was noted in the bodily pain, physical function and social function subscales of SF-36, both 6 and 12 months after surgery.

### 3.2. Functional Assessment

**Figure 2:**
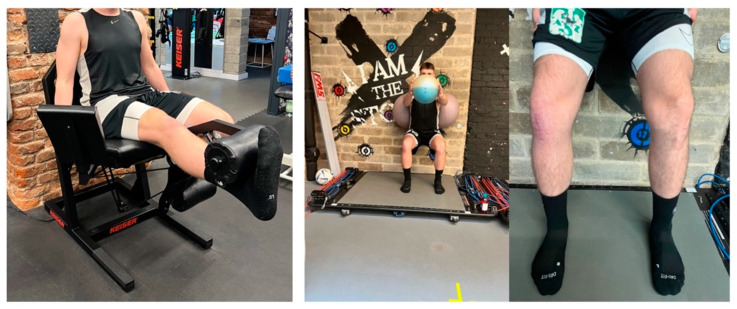
Professional soccer players during rehabilitation.

The patients resumed painless daily activities 3 to 4 weeks after surgery. The time taken to return to sports ranged from 2 to 5 months (Figure 2). All professional soccer players (100%) recovered to pre-injury levels of play (mean time 2.5–3 months). At the 6- and 12-month follow-up visits, we observed no anomalies concerning range of motion, edema, or discomfort in the treated joint. The findings indicated satisfactory joint function and no adverse effects associated with the treatment.

### 3.3. Radiological Evaluation

**Figure 3:**
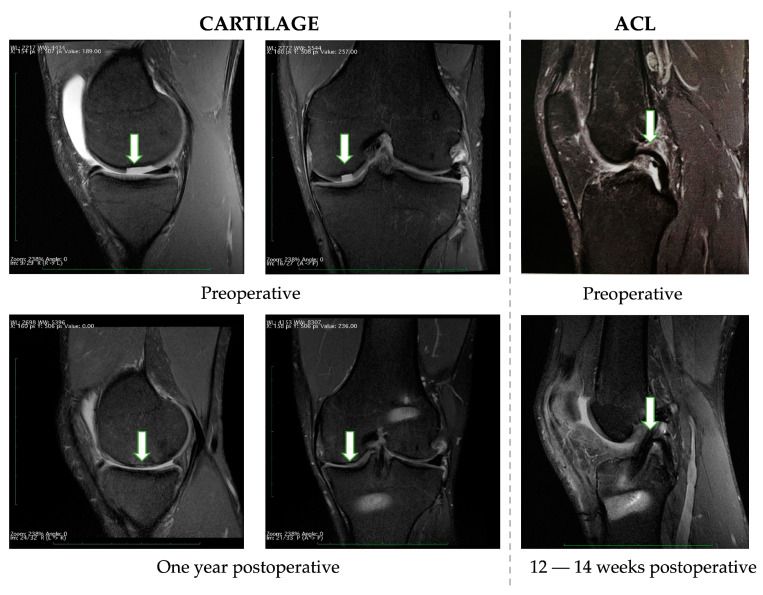
Preoperative and one-year postoperative MRI data demonstrating cartilage regeneration progression (the arrows show the cartilage defect and marrow edema before surgery, and the healed defect and withdrawn marrow edema one year later). 12–14 weeks postoperative MRI on the right side demonstrated full remodeling of the ACL graft.

Preoperative and postoperative MRI was performed in all patients. All the soccer players involved in this pilot study had similar results. Figure 3 shows an example of these results. When preoperative and postoperative MRI results of the cartilage are compared, the inflammatory reaction in the periosteum is no longer visible. Furthermore, a foreign body (a torn-out piece of cartilage) was evident prior to the operation but was no longer visible after the surgery. New tissues have been used to fill cartilage flaws. The subchondral layer edema subsided (showing healing of the wound). Based on observations carried out 12–14 weeks after surgery, healing of the bone blocks and full remodeling of the graft are already visible in the case of the ACL.

### 3.4. Complications

The study reported no postoperative complications such as infection, bleeding, liver/kidney dysfunction, or any other knee injuries. The absence of these complications further supported the safety and effectiveness of the treatment methods used in this study.

## 4. Discussion

This study’s main finding indicates that the combination method for treating defects of chondral tissue and ACL injuries, along with supervised rehabilitation, proves to be an effective and promising method for treating professional athletes. This comprehensive treatment approach leads to improved patient comfort, enhanced quality of life, and enhanced physical function, making it a favorable and viable option for athletes aiming for swift return to sports at the pre-injury level. ACL and cartilage tissue reconstruction are surgical procedures often performed in soccer players for knee injuries. The knee plays a crucial role as one of the most vital joints in a soccer player’s body and damage to the ACL or cartilage tissue can result in limited mobility and force the player to stop his sports career [36,37,38]. In this pilot study effects of follow-up after Hyalofast scaffold cartilage surgery combined with ACL reconstruction in the case of a professional athlete were presented. During this period, no treatment failure or recurrence of symptoms within the treated knee joint were observed.

The co-occurrence of ACL injuries and acute chondral defects is a widely acknowledged phenomenon in the existing literature [7,36,37]. Indeed, amidst the controversies surrounding the approach and selection of cartilage defect treatments, one undeniable fact remains: certain lesions necessitate repair to prevent further deterioration. Preserving the integrity and functionality of the affected joint is paramount, and appropriate treatment interventions are imperative to achieve this goal [38].

Returning to sports activities is the most important outcome in ACL surgery, being the most important motivation for people (not only athletes) who undergo surgical reconstruction [6]. Krych et al. [39], in a study of 224 patients, showed that cartilage damage grade II or higher affects delayed recovery. Sandon et al. [40] in their study found that cartilage damage negatively affected athletes’ return to soccer after ACL reconstruction [40]. Asano et al. [41] evaluated factors that influence knee pain after ACL reconstruction during a 1.5-year follow-up. A total of 70% of the 102 patients were reported to complain of pain. The highly damaged cartilage led to a higher degree of perception of pain. These findings were also confirmed by McAllister’s team [42] in 55 studied patients analyzed 3.6 years after surgery. Based on observations, this study identified the degree and location of cartilage damage as the two most important factors affecting pain sensation and recovery [42].

In recent years, various surgical techniques have been developed and improved to manage cartilage defects, but there is still debate over which method is most appropriate. Professional athletes are more susceptible to cartilage injuries than the general population, and these injuries often lead to osteoarthritis, which causes pain and decreases quality of life [35]. Arthroplasty is a common solution, but it is invasive and expensive, and the lifespan of an implant is approximately 15 to 20 years, making it suitable primarily for older people (over 60 years old) [29]. The use of an already appropriate treatment for cartilage defects, microfracture, reduces the time required to return to sports (8.6 months) compared to osteochondral allograft transplantation (9.4 months) and autologous chondrocyte implantation (11.6 months). This makes this treatment strategy particularly desirable in athletes who are under time pressure to return to sports, with the knowledge that further treatment may be required after the end of their careers or if treatment fails during their careers [43,44].

Numerous studies are available in the literature, including randomized controlled clinical trials, which confirm that the treatment of cartilage injuries with microfractures and scaffolds leads to satisfactory results compared to other procedures such as microfractures alone [45,46]. The review prepared by Mithoefer team encompassed 12 studies involving a total of 611 patients treated with microfracture, and the mean follow-up duration was 46 ± 6 months. Remarkably, 67 ± 7% of athletes exhibited good to excellent results following microfracture procedures [47]. Our research findings demonstrate that the incorporation of an additional Hyalofast scaffold has significantly improved outcomes in the short-term follow-up period. Autologous chondrocyte implantation has proven effective for the repair of articular cartilage in athletes, including soccer players. Historically, with first-generation techniques, the average duration until a return to soccer was 18 months. However, advancements in second- and third-generation implantation techniques, coupled with expedited and sport-specific rehabilitation protocols, have notably reduced the average return-to-sport time to 11 months [15]. Nonetheless, our research demonstrates even more accelerated results compared to these newer techniques.

The combined approach of microfracture and the application of the Hyalofast membrane has enabled a swifter return to sports such as soccer, without compromising the capacity for sustained competition over time. The study carried out shows that a combination of Hyalofast scaffold implantation and microfracture technique, together with supervised rehabilitation, is a safe and effective method to treat defects of chondral tissue and ACL injuries. The findings derived from this study are consistent with other investigations indicating that patients attain favourable clinical outcomes through the application of hyaluronic acid-based scaffolds within cartilage damage treatment methodologies [48,49,50,51]. This treatment can improve patient comfort, quality of life, and physical function, making it a suitable option for athletes who want fast returning to sport. The treatment can be applied to athletes from different disciplines, but continuous rehabilitation and training are crucial for maintaining their well-being. Ian Tan et al. published a paper that support our findings [52]. The study, which involved 46 patients with knee cartilage damage (grade IV), demonstrated that the use of Hyalofast together with the microfracture technique is a viable treatment option. In their investigation, all KOOS scores evaluated one, two, and three years after surgery showed a marked improvement compared to preoperative results. In this paper they also showed postoperative MRI scans that revealed cartilage defects filled with chondral tissue, and near-complete repair of the cartilage surface.

Physicians treating professional athletes with cartilage damage face challenges related to the duration of the recovery period, as well as the possibility of restoring pre-injury performance levels. In the present day, rehabilitation protocols have evolved to adopt more aggressive standards compared to the past. These modern protocols emphasize achieving full knee range of motion, immediate participation in activities, immediate partial weight-bearing, and functional training. The shift towards more assertive rehabilitation aims to optimize patient recovery and facilitate their prompt return to normal activities and sports [53,54]. Regarding the rehabilitation protocol after ACL reconstruction and osteochondral grafts surgeries, many concerns still remain about the timing of the use of a brace, and the return to sports activities [13].

Hurley et al. [44] in their study listed rehabilitation protocols for range of motion (39 studies) and weight-bearing (47 studies) after microfractures. Mostly, range-of-motion exercises were started during the first week after surgery. But the decision to allow partial weight-bearing is variable. While 55% of studies recommended starting weight-bearing during the first week after surgery, 36% waited until the 4th postoperative week. The inclusion of full weight-bearing usually began only after six weeks (53% of studies) or even after eight weeks (35% of studies) [44]. Della Villa et al. [55] in their research recommended delay of full loading of the treated joint, because using of autologous chondrocyte implantation requires time for the graft to mature and form new cartilage. Consequently, the treated joint is not allowed to be full weight-bearing until 3–4 weeks after surgery [55]. Furthermore, Hambly et al. [52] found that it takes at least 18 months for the graft to remodel and during that period the cartilage becomes more hyaline. Therefore, the author suggests prolonging the return-to-pitch time from 12 to 18 months [52]. However, nowadays professional sports, extended recovery period can cause athletes to miss one or even two seasons of play. Our treatment option, which eliminates this long recovery time before allowing full weight to be carried on the treated joint, may be more appealing. Della Villa [15] also confirmed that an intensive rehabilitation program can produce a better therapeutic effect and a faster and safer return to sport [15].

The proposed treatment method differs from other approaches reported in the literature. Some authors recommend restricting weight bearing on the affected limb for four, six, or even eight weeks, and using a range of motion (ROM)-limiting orthosis. In contrast, our approach involves early mobilization and full weight-bearing on the treated joint, with the use of crutches stopped just two weeks after surgery. This approach may seem radical, but our study shows that it does not negatively affect the cartilage regeneration process and allows patients to quickly return to sports and regain their independence. Patients were pain-free within 1–2 weeks of surgery, and pain elimination was sustained with no recurrence in all cases. Three weeks after surgery, patients commenced a pitch training program tailored to their specific sport discipline. This personalized training approach not only served to prevent the development of fear of re-injury, but also significantly bolstered their confidence in utilizing the treated limb. Notably, our longest follow-up period spanned 24 months, and throughout this duration, we observed no instances of treatment failures or recurrence of symptoms within the treated knee joint. The MRI results provided compelling evidence, as all defects showed complete healing with regenerated cartilage, and there was no evidence of periosteal reaction. These findings underscore the successful outcome of the treatment and the efficacy of the applied therapeutic strategy in promoting cartilage repair and patient recovery.

The study’s strengths lie in several key aspects of the research design and obtained outcomes. The study adopts a comprehensive treatment approach that includes ACL restoration, microfracture and hyaluronic acid scaffold cartilage repair surgery (comprehensive combination therapy approach). This combination therapy addresses both ACL and cartilage injuries simultaneously, providing a well-rounded treatment option for the patients. The study focuses on a specific and relevant population of professional soccer players, who often face challenging knee injuries that can significantly impact their sports performance and careers (including professional soccer players). By targeting this specific group, the study addresses the unique needs and demands of high-level athletes. The implementation of early supervised physiotherapy and complete weight-bearing immediately after surgery highlights the importance of early mobilization and intensive rehabilitation in promoting a rapid recovery and return to sports activities.

The results demonstrate that the patients resumed pain-free activities within 3–4 weeks and returned to their pre-injury level within 4.5 months. The MRI findings show release of marrow edema and the emergence of new cartilage, indicating successful cartilage regeneration. The KOOS and SF-36 questionnaire results indicate a significant improvement in patients’ health condition and quality of life at the six-month and one-year follow-up, further supporting the positive impact of the combination therapy approach. Overall, the study’s strengths encompass its targeted approach to addressing specific knee injuries in professional soccer players, the use of a comprehensive combination therapy, early and intensive postoperative rehabilitation, objective follow-up assessments, and the demonstration of positive outcomes and improvement in patients’ health and quality of life. These strengths contribute to the study’s scientific significance and potential impact in the field of sports medicine.

Our study has certain limitations, including the lack of a control group treated with a less intensive rehabilitation program. However, it should be noted that patients who are not professional athletes often do not exhibit the same level of motivation during the rehabilitation period. Furthermore, a randomized trial with a longer follow-up period could provide more data; however, such a study would likely be challenging to conduct. The study’s small sample size, involving only 12 professional soccer players, may limit the generalizability of the findings. A larger and more diverse cohort would strengthen the study’s statistical power and enhance the representativeness of the results. One of the limitations of this study was the relatively short-term follow-up period. While the data obtained during the study provided valuable insights into the early outcomes of the one-stage ACL and cartilage repair procedure, a longer follow-up period is necessary to assess the treatment’s durability and effectiveness over time. As mentioned, the study design was retrospective, which introduces inherent limitations in data collection and potential for bias. Retrospective studies may be susceptible to information bias and confounding variables that could influence the outcomes. Although we carefully analyzed and controlled for confounding factors, prospective studies with well-defined protocols offer stronger evidence. The retrospective nature of the study may introduce a risk of bias in data collection, outcome assessment, and patient selection. We acknowledge this potential limitation and have made efforts to minimize bias by ensuring consistent data collection and employing objective outcome measures.

Future investigations should consider the conduct of randomized controlled trials with extended follow-up durations should be contemplated. While inherently challenging, these trials could yield more robust and comprehensive datasets concerning the long-term ramifications of one-stage ACL and cartilage repair interventions. Prolonged follow-up periods are imperative for the assessment of treatment sustainability and efficacy across extended temporal dimensions. Expanding the cohort size to encompass a more extensive and heterogeneous population of athletes is paramount. Augmenting the study sample to include participants from diverse sporting disciplines and skill levels would amplify statistical robustness and bolster the generalizability of outcomes. The incorporation of athletes representing a spectrum of sports and proficiency levels would afford a broader comprehension of the applicability of the treatment modality. The continuous monitoring of patients over extended temporal horizons can illuminate the treatment’s performance characteristics and unveil any protracted complications that may ensue.

## 5. Conclusions

In this study, the efficiency of ACL restoration and cartilage regeneration was investigated in one-stage surgery treatment. The quality of life of the patients after surgery was improved by ACL restoration and Hyalofast membranes, as well as microfracture surgery and tissue adhesive. Patients were able to return to routine activities and sports more quickly thanks to the recommended treatment. A postoperative rehabilitation program is critical to optimizing cartilage defect repairs and reducing the time required to return to full athletic performance. The main finding of the study is that individuals with both a grade IV cartilage injury and ACL damage can be adequately repaired with simultaneous surgery under the same anesthetic and because of a highly intensive rehabilitation program, they can come back faster to sport activity at the pre-injury level. Clinical outcomes are satisfactory, and patients’ quality of life improves as compared to their pre-treatment status. The intervention program’s effect underscores the practical application of a comprehensive treatment approach, offering valuable guidance for healthcare professionals involved in the management of complex knee injuries. These findings emphasize the importance of personalized treatment strategies and intensive rehabilitation in optimizing patient outcomes.

## Figures and Tables

**Table 1:** Characteristics of the athletes included in the study.

	Knee Joint *n* = 12
Age, Mean ± SD	26 ± 4
Sporting activity	
Professional	12
Gender F/M	0/12
Cause of damage	
Traumatic	12
Non-traumatic	0
Location of the lesion	
LFC	0
MFC	12
Size of the lesion [mm^2^]	60 ± 12

Abbreviations: SD—standard deviation, M—Male, F—Female, LFC—lateral femoral condyle, MFC—medial femoral condyle, *n*—number of patients, mm^2^—square millimeters.

**Table 2:** Rehabilitation protocol used in the study.

Stage	Time Frame	Physiotherapy Treatments	Exercises
1	Depending on the individual progress and abilities of the patient, which typically takes around 1 week on average.	Learning to walk with crutches. Cooling Electrostimulation Lymphatic drainage Manual therapy, mobilisation of the patella Patient education Rolling of the operated and non-operated limb	Learning the proper walking Trampoline Exercises Active exercises to improve range of motion Mini squats and lunges
2	Depending on the individual progress and abilities of the patient, it typically takes between 2 and 3 weeks on average.	As in stage 1, without crutches. In addition: flossing	As above, by increasing the difficulty of the exercises, Stabilization and proprioception training using unstable ground Training with the use of elastic bands Exercises on one limb Full squat Muscle strength exercises Driving, life activities without restrictions Exercises on a stationary bicycle Exercises in a swimming pool
3	Depending on the individual progress and abilities of the patient, which typically takes around 3 to 16 weeks on average.	Treatments as described above and, in addition: Manual therapy Customization of treatments for specific conditions	Plyometric exercises (jumping and landing drills) Running and jogging training (initially, for example, with an antigravity treadmill or aquatic therapy) Coordination and speed exercises Resistance training with added weight Proprioceptive training with unstable surfaces Resistance band exercises Swimming Straight-line running exercises
4	Depending on the individual progress and abilities of the patient, it typically takes around 4 to 6 months on average.	Treatments as described above	Sport-specific exercises that are highly specialised in the discipline Continual development of strength, muscle control, proprioception, and active stabilisation Gradual increase in exercise intensity, such as adding new loads and increasing the number of repetitions Education of the patient about injury prevention principles Gradual re-introduction to the sport Participation in matches and competitions

**Table 3:** Comparison of the preoperative and postoperative results from the KOOS and SF-36 questionnaires for all patients.

		Preoperative	Six Months Postoperatively	One Year Postoperatively	Statistical Significance (*p*)	
	Parameter	Mean Value ± SD	Mean Value ± SD	Mean Value ± SD	(Before/Half a Year)	(Before/One Year)
KOOS Score	Patients (*n*)	12	12	12		
	Pain intensity	66 ± 13	98 ± 3	95 ± 7	**	**
	Symptoms	47 ± 16	95 ± 5	90 ± 12	**	**
	Activities of daily living	66 ± 19	99 ± 3	98 ± 4	**	**
	Sport/Rec	26 ± 9	91 ± 23	88 ± 23	**	**
	Quality of life	38 ± 18	70 ± 16	77 ± 14	**	**
SF-36 scale	Patients (*n*)	12	12	12		
	Physical Function	63 ± 28	90 ± 23	97 ± 3	*	**
	Role Physical	61 ± 40	71 ± 23	87 ± 17		
	Bodily Pain	47 ± 22	76 ± 10	84 ± 15	**	**
	General Health	72 ± 20	71 ± 9	70 ± 9		
	Vitality	62 ± 13	62 ± 13	68 ± 9		
	Social Function	60 ± 29	84 ± 19	89 ± 13	*	**
	Role of Emotional	60 ± 34	85 ± 15	90 ± 19		
	Mental health	55 ± 20	67 ± 9	67 ± 9		

Abbreviations SD—standard deviation, *n*—number of patients, *—statistical significance *p* < 0.05, **—statistical significance *p* < 0.01.

## Data Availability

The data presented in this study are available on request from the corresponding author. Data are not publicly available due to patient data protection.
